# Miscellaneous

**Published:** 1874-08

**Authors:** 


### MISCELLANEOUS.

### CHOOSE—HEALTH OR SICKNESS.

THOSE who desire and appreciate health, should be as willing to make some effort to secure it as they do to obtain the other good things which increase the pleasure of life. Pure water is essentially necessary to good health.

All wells, cisterns and springs should be thoroughly cleaned in the early spring, or in the autumn. The usual method of placing a large stone on the top of the cistern is injurious to the water, unless an aperture is left in the stone and fitted with a wooden cover. The air should not be wholly excluded from the cistern, else mouldy conditions will predominate—although perhaps not apparent—and the water will not be wholesome, and in it sometimes there may be found various kinds of insects and reptiles. Water is the natural drink of all living creatures, and "it serves several important purposes in the animal economy—firstly, it repairs the loss of the aqueous part of the blood caused by evaporation, and the action of the secreting and enhaling organs; secondly, it is a solvent of various elementary substances, and therefore assists the stomach in digestion, though, if taken in very large quantities, it may have an opposite effect, by diluting the gastric juice; thirdly, it is a nutritive agent—that is, it assists in the formation of the solid parts of the body." All the water used for domestic purposes should be first subject to filtration; it will remove the impurities and "animal and vegetable substances which are not held in chemical solution." It is very injurious to drink water which contains impurity in solution, as no process will then purify it, and very wrong to use it for domestic purposes because it is useful for other purposes. "Health is economy," sickness the reverse.

"It is impossible to enumerate the various substances which, to a greater or less extent, enter into and affect the character of water after it reaches the earth; therefore, cistern water can be made the most pure of any kind of water—although cisterns can be penetrated at great distances by offensive vaults or other impurities." "It is sickening in most country places to observe the uniformity with which the well and a cesspool are made to stand side by side, as though each was necessary for the other." Sickness would be the "exception and not the rule" if all persons were as anxious to know the conditions which promote health and longevity as they are to acquire unnecessary wealth.

*Leavenworth, Kansas.* V. E. L.

### THE CAUSES OF DECAY OF TEETH.

It has been charged against our brethren of the dental specialty, says the *Lancet*, that they are wofully at fault in regard to knowledge of the commonest of all things—caries of the teeth. That they extract teeth with skill, and stop them with even more skill, and in a nobly conservative spirit, is admitted; but the causes of decay in the teeth have remained obscure. The investigations of Leber and Rottenstein into this subject have at least the charm of pointing to definite conclusions. They admit, of course, that there are differences of teeth, constitutional and connected with race, making teeth more or less resistant to the great influences which determine decay. These are not, according to these authors, internal and vital so much as external and chemical. The process of decay begins from the surface, and if it can be controlled or arrested at the surface, it is entirely controlled. The great causes of caries are two, namely, acids and certain fungus found abundantly in the mouth, *leptothrix buccalis.* This latter agent is characterized by certain microscopic appearances and by its reaction with iodine and acids, which give to the elements of leptothrix a beautiful violet tinge. Under the microscope the fungus appears as a gray, finely granular mass or matrix, with filaments delicate and stiff, which erect themselves above the surface of this granular substance so as to resemble an uneven turf. The fungus attains its greatest size in the interstices of the teeth. No one can deny nowadays the action of acids on the teeth, even weak acids, in dissolving the salts of the enamel and the dentine. All acids, both mineral and vegetable, act promptly on the teeth. Various experiments as to the action of acids on dental tissues are given, making the enamel, naturally transparent, first white, opaque and milky, and, in a more advanced state, chalky, and then the dentine more transparent and softer, so as to be cut with a knife. The acids which may actually effect the first changes in the production of caries are such as are taken with food, or in medicines, or such as are formed in the mouth itself by some abnormality in our secretions, which should be alkaline, or by an acid fermentation of particles of food. But acids alone will not account for all the phenomena of caries in the teeth. They play a primary and principal part, making the teeth porous and soft. In this state, the tissues having lost all their normal consistency, fungi penetrate both the canaliculi of the enamel and of the dentine, and, by their proliferation, produce softening and destructive effects much more rapid than the action of acids alone is able to accomplish. It is not pleasant to think that fungi exist in the mouths of all but the very cleanest of people. Bowditch, in examining forty persons of different professions, and living different kinds of life, found in almost all vegetable and animal parasites. The parasites were numerous in proportion to the neglect of cleanliness. The means ordinarily employed to clean the teeth had no effect on the parasites, while soapy water appeared to destroy them. If this be a true version of the causes of caries— the action of acids, supplemented by the action of fungi—then it follows that the great means of preserving teeth is to preserve the most scrupulous cleanliness of the mouth and teeth, and to give to the rinsing liquids a slightly alkaline character, which is done by the admixture of a little soap. This is not so pleasant a dentifrice as some, but it is effective and scientific. Acids not only dissolve the salts of the teeth, but favor the increase of the fungi of the mouth. No increase of fungi and no action on the dental tissues occurs in solutions slightly alkaline, such as a weak solution of soap. The good effects of stopping teeth, in the light of these experiments, are intelligible. The penetration of acids and fungi is prevented.

Peter Kalm, the Swedish writer, who described in a very intelligent manner what he saw and heard during a trip through the United States, observed a frequent loss of teeth among settlers from Europe, especially women. After discussing and rejecting many modes of explanation, he attributed it to hot tea and other hot beverages, and came to a general conclusion that "hot feeders lose their teeth more readily than cold feeders."

Mr. Catlin, who some years ago gave, in England, an interesting exhibition of Indian scenery, dresses, weapons, etc., noticed that North American Indians have better teeth than the whites. He accounts for the difference in this strange way—that the reds keep the mouth shut, whereas the whites keep it open. The teeth, he says, require moisture to keep their surfaces in good working order; when the mouth is open the mucous membrane has a tendency to dry up, the teeth lose their needed supply of moisture, and thence come discoloration, toothache, tic doloreux, decay, looseness, and eventual loss of teeth. Mr. Catlin scolds the human race generally "for being less sensible than the brutes in this respect, and the whites especially in comparison with the reds. We keep our mouths open far too much. The Indian warrior sleeps, hunts and smiles with his mouth shut, and respires through his nostrils. Among the virtues attributed by him to closed lips one is excellent—when you are angry keep your mouth shut."

It would certainly be a decided gain to humanity if the Mrs. Caudles and juveniles could be made to believe that much opening of the mouth tends to ruin the teeth.

### LEAD PIPES AND WATER SUPPLY.

The question whether water is poisoned by flowing through lead pipes was lately discussed in the French Academy of Sciences, with results that are calculated to quiet the apprehensions of those who get their water supply from such pipes. M. Dumas stated that in his chemical lectures he had long been accustomed to employ a very simple experiment for the purpose of showing that water corrodes lead only under special conditions. He takes distilled water, rain water, spring water, etc., and drops into each pieces of lead. It is found that only the distilled water acts on the lead, the salts of lime in the rest of the specimens preventing the reaction. M. Belgrand read to the Academy a memoir giving the results of his investigations into this subject. The ancient Romans employed lead water pipes on a large scale, but yet no Latin medical writer says anything of lead poisoning produced by the water. According to M. Belgrand, one sixth of a grain of calcareous salts to the quart prevents the dissolution of the lead. He exhibited to the Academy pieces of lead pipes which had been in service from the time of Louis XIV without showing any sign of corrosion ; and analysis of water that has passed through a long line of lead pipes showed the complete absence of lead.

### HYDROPHOBIA.

At the meeting of the French Academy of Sciences, on April 13, M. Bouley laid before it a memoir by M. Bourrel, a veterinary surgeon of Paris, entitled a "Complete Treatise on Rabies in the Dog and Cat, with a method of Preserving one's self against it." The means of preserving from rabies, to make known and disseminate the knowledge of which is the principle aim of this memoir, consists in taking off the edge of the teeth of the dog by the aid of nippers and files. M. Bourrel had the daring to perform this operation of filing down the teeth on three dogs when they were in a condition of raging madness, notwithstanding the danger of inoculation he incurred both during the preliminaries and the different stages of the process. Six dogs kept for experiment were then delivered over to the mad animals, who precipitated themselves on them and bit them furiously, but without breaking the skin in any one of them. The dogs experimented on were watched during six months, and madness did not show itself in any of the number. M. Bourrel, convinced that the blunted tooth of the dog could not penetrate through clothing, gave his hand, covered with a glove, to one of the mad dogs. "When," he says, "the dog released it, the glove was intact, and the bite had only produced a deep impression." This experiment, repeated on dogs who were not mad, to which I gave my naked hand to bite, proved to me that the blunted tooth can but very rarely, however great may be the contraction of the muscles of the jaw, break the epidermis of animals, whose hair necessarily deadens the pressure exerted, and can only injure the human epidermis in very exceptional cases.

### DYSPEPSIA OR BARBARISM.

During the last day of the session of the State Medical Society, one of the members, while referring to the remarks made by the President of that body at the banquet the previous evening, relating to the American style of eating, related an incident that had come under his observation at the breakfast table of the hotel where he was stopping, which showed, he said, the close connection between dyspepsia and barbarism. The doctor proceeded to say that, upon taking his seat at the table, his attention was attracted to two gentlemen who were seated opposite to him, one of whom made some inquiry in regard to the health of the other. The reply returned was: "I am not feeling very well; I am suffering from dyspepsia." At this juncture a waiter appeared upon the scene, and placed before the dyspeptic gentleman his breakfast, which consisted of three boiled eggs, two baked potatoes, a plate of beefsteak, a cup of coffee and four buckwheat cakes. The doctor was just then in the act of winding his watch, and concluded to time the victim of dyspepsia, who startled him by bolting all the edibles set forth in the remarkably short space of two minutes, ten seconds. "Now," quoth the doctor, by way of concluding his story, "was this a case of dyspepsia or barbarism?"

### THE VALUE OF NOURISHING FOOD.

It was owing, in a great degree, to the wretched condition of their commissariat that the Austrians were defeated at Austerlitz. *C'est la soupe que fait le soldat.* "Let us eat and drink, for to-morrow we may die," is a motto which has often been denounced, and most justly, by Christian moralists. "Let us eat and drink well, lest to-morrow we die," would be a good substitute. The pleasures of the table are not the highest form of human enjoyment, it is true; but for all that, an oyster pie is a good thing when well made. "A man," says Dr. Johnston, "who has no regard for his stomach, will have no regard for anything else." We fully agree with the great moralist, and we subscribe no less heartily to the saying of the French gourmand, who declared that the discovery of a new dish is more important than the discovery of a new star, because there never can be dishes enough, but there are stars enough already. It is a mistake to suppose that only brainless men, with full paunches and empty pates, have a keen relish for the luxuries of the table—that, as Shakspeare says,

> "—— Dainty bits
> Make rich the ribs, but bankrupt quite the wits."

The celebrated scholar, Dr. Carr, confessed a love for "hot lobsters, with a profusion of shrimp sauce." Pope was a decided epicure, and would lie in bed for days at Bolingbroke's, unless he were told that there were stewed lampreys for dinner, when he would rise instantly and hurry down to table. Cleopatra is said to have owed her empire over Cæsar as much to her suppers as to her beauty; and who can tell how much the love of the Grand Monarque, Louis XIV, for Madame de Maintenon was owing to the invention of the immortal cutlets which bear her name. Cardinal Wolsey was conciliated by the good dishes on the Field of the Cloth of Gold; and Agrippina won Claudius by a receipt for dressing Spanish onions. Handel ate enormously; and, when he dined at a tavern, always ordered dinner for three. On being told that all would be ready as soon as the company should arrive, he would exclaim: "Den bring up de dinner prestissimo. I am de company."

### NOT LOST.

Love is all things in one to us. It is hope, and fear, and joy, and despair; it is truth and it is falsehood; it is anything, in fact, that you are pleased to call it, and it can represent the brightness of heaven or the blackness of perdition.

"Love is a melting of the soul."

It was late in the afternoon of a dull autumnal day that a group of young people came chatting down the flight of stone steps leading from the door of a cathedral church, in an old Atlantic seaport town. They were members of the choral society attached to the church, and they had evidently been there for rehearsal. Within, the great building yawned black and lonely, save in the gallery, where, over the organ, a gas jet spun rays of light in the gloom, and the sound of softly subdued voices broke through the stillness.

The visible occupants were two, a man and a maiden—young, and with the cabalistic word "lovers" gleaming, as did the handwriting of old on the wall, on their foreheads. Robert Field, the organist, was turning over some sheets of manuscript music with an absorbed air, while by his side stood Hester Heathersleigh, her pretty face full of anxious interest as she watched his movements. A little cloud of uneasiness wrinkled her forehead now and then as she saw the rent edges of angry clouds scud by the narrow slit of window opening to the east, where the gray sea lay tossing stormily.

"Well, Robert!" she said at last, dropping her slim hand on his shoulder. "Well, Robert, what is it?"

The musician's dark, serious face lighted a moment gloriously as he turned and took the little ungloved hand in his.

"I asked you to stay, Hester, because I wished to play for you some passages from my new piece. I shall submit it to the society at Music Hall to-morrow evening, and I want your opinion in advance."

The young girl laughed—a little, rippling laugh of gleeful enthusiasm.

"My opinion! Why, Robert, you know beforehand what that will be. It would be nothing but a form asking it."

Robert raised the little hand tenderly to his lips.

"I know that love makes gentle critics of us all," he said, wisely. "But now I want you to forget who is the author of the melody, and to exercise your judgment without stint. Remember, too, that love is the theme; love which, wisely or unwisely, hopes all things, believes all things, and endures all things unto the end." And then he turned to the organ.

He played slowly at first. It was a lonely opening, full of strange sad chords, as if a soul were waiting somewhere in shadow. Then, as brightness entered, the theme asserted itself. The wonderful tones climbed higher and higher, expressive of a great faith, of a fond, mad triumph and bewildering joy. On and on the chords swept; it was as if a living chain of light ran round the world.

When he had finished there was silence for a moment between these two. The lingering echoes rolled back and forth till one by one they, too, escaped into stillness. Then Hester Heathersleigh stooped, and, with quivering lips and tear-wet eyes, reverently kissed the bowed forehead of her lover.

"Oh, my darling!" she cried, "it is so beautiful! I am so proud of you. Who taught you to play like that?"

A proud and satisfied smile curved Robert Field's lips as he listened. "My love for you taught me," he answered. "My love for you, which is so great, so all-absorbing, that my music seems to be but a poor expression of it."

Then lifting her head he gazed for a moment with wistful tenderness into the rose-pink beauty of her small, sweet face.

"You think it is a triumph, then, dear? Ah, Hester, are you sure you speak for the music itself, or only out of a tender mercy born of your love for me?"

An indignant light brightened the pretty violet eyes out of the drowsy languor of youth's enchanting dreams.

"Tender mercy for you," she repeated. Then her voice changed. Ah, Robert! if my love can make you write like that now, then your future life shall be full of inspiration, for I shall love you more and more the longer I know you. I shall love you more and more forever."

She wound her arm about his neck, and with tender, maiden sweetness kissed his forehead, and kissed his wavy hair, and kissed the thin, pale hand which lay nervelessly on the yellow organ keys. And then a stillness crept about them, a stillness more fraught with eloquent joy than any measure of golden speech could have been.

While they thus stood hand in hand talking, the curtain behind them partitioning off the long gallery parted, and a dark face peered through. It was a man's face, handsome but cruel in that purple gloom of gathering shadow. It was no friendly face, either, that with its many changes of hate, and jealous anger and furious despair, seemed, while the lovers talked, to be playing a dark and stormy accompaniment to the idyl of their love.

A sudden angry burst of wind at the narrow window roused them unpleasantly to a sense of night and the nearing storm.

"Oh, the rain," cried Hester, with a pale face. "How thoughtless of us to stay, and you have that long, desolate walk over the cliffs in the dark!"

"Never mind," cried Robert, stoutly. "There are such light and warmth within me that I shall not heed a passing touch of wind and water. I will see you to your door first, and then good night."

"My cousin Conrad promised to come for me," Hester answered. "I wonder what detains him. It is too bad for me to take you all this long way out of your route."

"I like it better so," the young man said, gravely. "I do not like your cousin Conrad, and I am not willing to trust you to his care. Oh, my darling," he went on earnestly, "if my music but brings me fame and fortune I can then make you all my own, and there will be no more good nights, no more partings in the storm for us."

They passed down the stairs and out into the street together, unconscious of the shadow closing upon them, nearer and blacker. At the door of Hester's home they parted with a lingering good-by.

"My precious music," cried Robert, buttoning his coat closer about him. "No harm must come to that. It represents fame, and fortune, and love and honor for thee and me, my darling."

Hester lifted a small wet face to peer into the gloom.

"I wish you could stay," she said. "And, oh! Robert, be careful of the cliffs—the path is so lonely and dangerous. I

shall come early to rehearsal to-morrow, for the sake of knowing that you are safe."

"Do," he answered. "I shall bring you glad tidings. Success is too near for me to miss it now. Good night, good night, my sweetheart." And so speaking, he passed from her into the shadow of his waiting doom.

After that night of storm the day dawned clear and cool. At St. Paul's the Choral Society, just then in the first flush of enthusiasm over a new oratorio, gathered early. One—two—three! the great bells chimed the hours, and the singers waited impatiently for their leader. Something had detained him, most likely—he would come soon. The hour struck four and he had not come, and Hester Heathersleigh, with a heart heavy as lead in her bosom, fell on her knees in an agony of prayer. "Oh, my God!" she cried, reckless of who might hear her, "he is dead! my Robert is dead! He has been lost in the cruel storm!"

Some one, pitying, touched her arm. It was her cousin, Conrad Charteris. He was looking down at her with a pale face—a face paler far than that with which he had spied upon her yesterday from behind the gallery curtain. Her piteous cry had touched even his stony heart. "Hush!" he whispered, "here is news from him—from Robert; come and hear what it is."

A note had been brought by a swift running messenger, and a shudder ran round the waiting circle of listeners when its contents were made known. It was signed by a leading physician of the city, and stated that Robert Field had been picked up that morning at the foot of the cliffs and taken home for dead. He was now, at the date of the writing, lying in an insensible condition, and it was impossible to tell what the extent of his injuries were, or if there were any hope of his ultimate recovery.

A horror stricken silence followed the reading of the note, broken at last by a low, sobbing cry from Hester Heathersleigh's white lips.

"I must go to him—oh, I must go to him! Who will take me? You! you!" and she caught Conrad Charteris by the arm.

He shrank away from her with a gesture much as if she had pierced him with a knife. His black eyes dilated horribly.

"I? I go with you to see him?" he cried. "What are you thinking of? What do you take me for?" Then noticing her astonished look, he made a fierce struggle for composure, but his hands shook like withered leaves.

"Why do you wish to go to him?" he questioned, angrily. "He would not recognize you—and it is no place for you! Let me take you home."

She snatched up her shawl and bound it with trembling fingers about her shoulders. "I tell you I shall go to him," she answered. "I was to have been his wife, and, living or dead, my place is now by his side. You can come with me if you like!" And she flew down the steps.

It seemed an age to her, that short time she was on the road leading to the lonely house of Robert Field's widowed mother; and when at last, by dint of her prayers and tears, she was suffered to approach his bedside, she looked down on a very different Robert Field from the one with whom she had parted in such high hope the night before.

The bruises were chiefly about the head, the physician said gravely, and even if he recovered it was doubtful if his mind would ever be sound again. Hester heard him, and with a great sob fell on her knees by the bedside. Where now were the brilliant aspirations, the tender hopes, the gay courage and stout-hearted faith of one short day gone by? Lost! lost! Success so near to him, and yet to fail. Triumph so nearly won, and yet to pass by on the other side.

"Robert, O my Robert! Look up! Speak to me, or I, too, shall die!"

Ah! but love remained. Love unchanged and unfaltering. This then was left—the blessing of a love which believes all things, hopes all things, and endures all things unto the end.

The drawn white face on the pillow did not change at Hester's cry, but under the half closed lids the dull eyes gleamed feebly, and the slender hand outside on the coverlet groped helplessly. Hester took his hand in hers, and then, quick as lightning, by some strange, subtle instinct, rather than by any demonstration of his, she felt that the poor, stricken senses were trying to break through the darkness that enveloped them and make their unknown want understood.

"Robert, Robert! what is it?" she cried. "What is it that you want to make us understand?"

The helpless movement of his lips, the helpless groping of his fingers, were enough to make one weep. Hester bent her ear to his mouth.

"What is it, Robert, dear? Tell me; what is it you want?"

The stiffened lips strove with a terrible effort to move, and this time one word was freely articulated:

"Music!"

Hester looked up with a startled exclamation.

"Music! He calls for music. Do you not hear? Where is it? Who knows about it? Is it lost?" she questioned, eagerly.

Again that terrible attempt at speech. The dull eyes opened wide, the feeble fingers clenched themselves on Hester's hand, and with a last mad effort of expiring desperate strength, he raised himself and shrieked:

"My music! Find it! Save it!" and then he fell back on his pillow like one dead.

"You have killed him," said the physician, angrily, and at the words Hester, with a moan, dropped down insensible.

Not dead! But when, after weeks and months of painful illness, he faced the world again, he looked like a shadow out of the past. But bent and aged, with scarred forehead and whitened locks, the wreck of his body was not the greatest evil that had befallen him, for of the brilliant genius of other days no vestige was left. Saddest of all, the miserable ghost of his lost hopes haunted him, and in the ruined chambers of his darkened intellect he was forever groping, trying to gather up the mystic chords of tuneful thought which no longer vibrated to his magic touch. The lost manuscript music had never been recovered, and though his feeble mind failed to take in the greatness of his loss, the shadow of something beautiful which was to have been, but, somehow, failed to be, lay on him, and gave his face a wistful look, which was sadder far in its mute endurance than any wail of speech could have been.

Music was to him now something akin to the sound of "sweet bells jingled out of tune and harsh."

One day in early spring he went to the church for the first time, leaning on Hester's arm. The old, familiar look of the place struck him forcibly and roused his dormant wits. He sat down to the organ and glided his hands over the keys; a few jangling discordant chords followed, wandering and disconnected; then his face changed, and, with a terrible cry, he flung his head down on his arms.

"Oh, Hester! tell me what it is I have lost! Sometimes I almost reach it—it is in my mind; something beautiful, which I almost grasp, and then it eludes me and fades away. I have lost it now. Hester! Hester! take me home!"

She kissed him and soothed him with sweet womanly words, and when he was more composed she led him away.

Soon after that they were married. In vain Hester's friends threatened and oppressed her. She was quietly determined.

"He loved me when friends and fortune smiled on him," she answered them. "He would have given me every great gift which the world was ready to bestow on him for love of his beautiful genius, and shall I desert him now when misfortune has overtaken him? Perhaps—oh, perhaps some time God may restore to him his lost mind!" Tears filled her lovely, soft, pathetic eyes. "If I dared to hope it—oh, I'd give my life to have it so."

The day before her wedding she received a visit from Conrad Charteris.

"It shall not be!" he cried out vehemently. "Do you realize what you are doing? Why you had better far die at once, for Robert Field is but little less than an idiot."

"And if he were an idiot," returned Hester, bravely hiding her hurt at the brutal words, "even then would I marry him. I love him, and if not one vestige of his glorious intellect remained, I would be Robert Field's wife—and a proud one, too!"

"I believe you would," answered Conrad, looking with a fond, mad longing into the small, pale face, lifted so undauntedly to his dark gaze. "Hester, you will drive me mad. I would to heaven that Robert Field were dead. Why did he not die that night last winter?" and he struck his hand furiously on the table in a blind frenzy of despair.

"God knows it was from no lack of purpose in you that he did not die," retorted Hester, spiritedly.

She spoke at random, but Conrad shrank away with a white face. The idle words evidently hit him hard. They cut close and sharp as steel in their unexpected descent, and wheeling abruptly about he left her, and did not seek her again.

They were married quietly, and after that, in the tender security of her modest home, under the fond and cherishing care of his wife, health and strength came slowly back to the shattered frame of Robert Field.

Slowly, too, out of the darkness he begun to wrench, one by one, the secrets of his prisoned mind. Old melodies began to shape themselves under his touch, discordant and fragmentary at first, but gradually assuming symmetry and power.

"Not quite a wreck!" he would sigh, wistfully. "Some day some good genii will unlock my prison door and set me free."

In the child that was born to them, a beautiful boy, who sang sweet music in every tone of his childish voice, his pride was great. He talked of him, listened to him, watched him, and dreamed of him, predicting a future of which Bertrand was to be the perfect flower, the very golden rose of joy. So the years passed, and sweet Hester Field's fair face grew heavenly beautiful to see, with its tired look of patient waiting. God only knows how her heart failed her now at times, or with what fierce power she wrestled with her growing doubts, and prayed for strength to help her bear this cross, whose shadow fell even darker and deeper on her young life.

Had her love, then, been a sacrifice in vain?

But one day the answer came!

Returning one afternoon from a long walk, Robert Field stopped in the hall, spell-bound by the triumphant strains of some new and beautiful melody floating through the rooms. His worn face flashed with the old light of inspired thought; his eyes dilated; his whole form shook with a mysterious emotion.

"What is it? what is it?" he asked of his wife, who came to meet him.

"Bertrand's music," answered the proud mother, Hester

"He has been engaged with it a long time. He meant it to be a surprise for you."

Robert Field threw up his arms with a joyful cry.

"It is mine! mine! My lost music! the music I played for you that long forgotten day! Hark, Hester! do you not recognize it now? Oh, to think that it has slept so long and now comes back to me so fresh and fair. This is what I have missed out of my life. This is my treasure, which was lost to me and now is returned to me after many years—brought back by a little child! Our child, Hester! Oh, thank God for that!"

Rushing into the parlor he swept Bertrand from the stool, and seating himself at the organ, with one powerful sweep of his hands over the keys he summoned his God-given genius from the tomb of his youth and bade it stand resurrectionized in new life before him. On and on the music swept; not a note was lost; not a chord dropped out of the splendid work. Shoutingly, exultantly the tones leaped forth, "and their name was called Wonderful." On! on! Up! and up!

At last, from sheer exhaustion, the musician dropped to the floor, and lying there at Hester's feet he wept tears which were no shame to him.

"It is the very same!" he cried. "Bertrand has written it out note for note, a counterpart of my own work. Is it not an awful thing to think of? My own work and yet his! Who but God can explain it? And oh, Hester! The darkness is all gone now! Let me thank God for that."

Then, wrapping his arms about her, Robert Field kissed his wife's pale face, and kissed her tender mouth, her wavy hair, and her slim, pale, faithful hands.

"My wife! my wife! Oh, what if your love had failed you, Hester? If, in those terrible first hours of my misfortune, your true heart had been one whit less true, then I should have been lying in my grave to-day, a broken and forgotten man!"

So fame and success, in the later days of his life, came, not unwelcomely, to Robert Field. The world welcomed his famous piece with none the less acclaim for its long delay, and for the strange story which accompanied it. One truth only concerning that fatal night Robert withheld—known alone to his faithful wife. But Conrad Charteris had long ago disappeared from the town, and was seen no more among them. So he and Hester buried the secret in their hearts, contented that it should be so—for God is his own avenger!

They had been taught a wonderful lesson, too, by One who, having lived on earth, knew what the full fruition of earthly life must be, and who gave, ere He passed away from among men, the crowning blessing of His wisdom in a last new commandment:

"Love ye one another!"

### ONLY A WOMAN.

Only a heart forever at rest,
Pulsing no more in the quiet breast;
Over the pale, cold lips no breath
Flutters back from the realms of death;
Only two chill hands crossed above
The quiet bosom, is all of earth
That is left of he whom I loved.

I loved, but my love will ne'er be known;
Only a woman, I could not ask
For that I'd have given my life to own!
Though, might I win him, this were my task—
To sit beside him so calm and pale,
To hide my love with modesty's veil,
To rest at ease 'neath his words of grace,
To glance at times in his calm, proud face,
And know that my heart beat only for him!

O Lord, thou has set me a harder task!
To bend o'er this still, cold form—and now,
Even now I wear society's mask,
That they who watch may see no tears flow.
O Father, no prayer of mine e'er may
Win the soul back to this lifeless clay
That lies before me in death's embrace.
My Saviour! have mercy, and give me grace
To carry my burden alone through life.

### NEW LEGEND OF THE "FORGET-ME-NOT."

WHEN Psyche lost her lord, the Lord of Love,
  Weeping alone she wandered
Listless by every well known field and grove,
  And on her lost love pondered.

Lastly by Lethe's stream her footsteps strayed;
  And "Oh!" she said, in sighing,
"That I might dip, and my past life be made
  Like dreams with daylight dying!"

The big tears from her blue eyes raining down
  Fell on earth's pitying bosom;
Sudden there sprang amid the sedges brown,
  Blue as her eyes, a blossom;

And o'er her head, soft rustling, sweet and low,
  As though some bird's wing fluttered,
In those loved tones whose loss was all her woe,
  "Forget-me-not!" was uttered.

No more; no sight, no touch; these words alone;
  And "Ah!" she cried, "forget thee?
Nay, but half love in our glad life was known;
  Half love to regret thee.

"Forget thee? Nay, these flowers my tears begot
  Shall be to me a token
Of love; they shall be called Forget-me-not!
  The name to cheer me spoken."

So well, sweet river-flowers, we welcome you,
  Earth with faint sadness scenting—
Born of the tears from Pysche's eyes of blue,
  For her lost love lamenting.

### THOUGHTS ON BEAUTY.

BY C. H. C.

IT is my belief that the plainest face may be made to grow beautiful, if, indeed, it possesses no positive deformity. I have seen faces without one regular feature—faces which in repose were undeniably ugly—render themselves, by a smile or an expression of the eye, irresistibly fascinating. This miraculous change of countenance was, of course, produced by the beautiful soul enshrined within—true beauty proceeding altogether from the soul—a beauty that never fades, never dies. How often do we see the most regular and statuesque features, combined with delicacy of complexion, wanting entirely that peculiar and irresistible charm, a lovely expression. There is no fair face so charming as that which is unconscious of its own beauty. I am sorry to say such a face is a *rara avis*, for almost every handsome man or woman is perfectly well aware of his or her attractiveness, and the simple consciousness of it gives to the countenance an expression of vanity and self-conceit.

I believe that it lies within the power of every human being to become, in a greater or lesser degree, the possessor of a soul beauty which will remain even in the countenance of age; it is simply to cultivate those graces and beauties of mind and disposition that are most calculated to give pleasure and happiness to others, as well as contentment and serenity to one's self. Meekness, modesty, innocence and singleness of heart, truthfulness and purity of soul, benevolence, and, above all, piety—each yields its own peculiar charm to the expression which glows in every feature, and especially in the eyes. A face of perfect beauty, like that I have in my "mind's eye," not only smiles with the lips, but the lovely soul shines out through every lineament, and actually sheds a halo around. Once or twice in my lifetime I have seen such a countenance.

I once knew a young girl, tall, fair and fragile as a lily, whose expression was the most angelic I ever beheld. It must have been one of the purest and loveliest of souls that animated that delicate form. Modesty and goodness beamed in her gentle smile, innocence and holiness dwelt in her sweet eyes of Heaven's own blue. She walked through the world like a being from a higher sphere—all reverenced, all worshipped her. Her beauty was not of earth, so she took her departure to the courts above, just like the melting of a snowflake—aye, just as silently and as yieldingly as a snowflake melts in the dark waters of the sea. For years consumption, like a "worm in the bud," had, almost unknown to herself, preyed secretly, but with a grasp, alas! too fatal, upon her delicate constitution. And, although neither herself nor her family appeared to observe it, strangers remarked with pain her gradual decline, and whispered their regret that one "so young and beautiful should perish with the flowers."

An angel *was* she—pure, confiding,
An angel *now*—more bright!
Too pure for earth—she's now abiding
In regions of delight.

### HEALTH RESORTS.

### THE BERMUDAS.

AMONG the near-by places of late years sought by invalids are the Bermudas. The climate of these islands is mild and of unusual evenness throughout the year, and is very beneficial to delicate or diseased lungs. Bermuda is only some six or seven hundred miles from this continent, and shares with Cuba and Nassau the advantage of being easily reached. It is not every one that can get as far as Madeira, or Italy, or the south of France. The preparation for any of these farther pilgrimages is much more elaborate and formidable. One can safely go to Bermuda without having so varied a stock of clothing, or so many trunks to hold it. No circular notes or letters of credit are required or could be used, but a few eagles and half eagles will safely land the tourist, and keep him from starving until the return of the mail boat from New York. The communication between Bermuda and New York, for some years, has been once in every three weeks by steam vessels, and at odd and intermediate times by sailing craft.

It is a most delightful change to leave the snow and cold of New York in December, and in a few hours to land on islands

### WHERE FLOWERS ARE IN BLOOM

and green peas are growing in the open air. If Bermuda lacked all other charms or attractions, its soft and safe climate would make it a haven for the sick and weary. The thermometer seldom gets as low as fifty, and, when it does get there, returns as soon as possible to its staying place among the seventies and eighties. There is no frost to check or kill vegetation. In the traditions of the island there are one or two occasions, with long intervals between, when a snow cloud has been blown over from our shores, and before it could get thawed or melted, has shed its sudden and startling chills over the islands. But such instances are very rare. The prevailing evenness of temperature does not vary twenty-five degrees from January to December. People with diseased lungs, or with fractions of lungs, or with no lungs to speak of, find comfort and delight in breathing the balmy atmosphere of the islands. It is just the place for those to whom, like the old lady in Colman's comedy, air is the chief attraction of the country. Air of the best and purest kind envelopes and bathes the islands. It comes directly from the Atlantic Ocean, untainted and without defilement, its only peculiarity being a salt strength, which may not suit all weaknesses or conditions of health. The whole length of the chief islands, strung like beads together, is not thirty miles; their greatest width at any point scarcely reaches the tenth part of their length. They form

A SORT OF TOY GROUP, and are sample bits or pieces of territory that serve to indicate the larger doings of creation in other parts of the world. It is some consolation to know that there is as much of them under water as there is above. All round the land are sunken reefs, which extend into the sea for distances as great, if not greater than the dry surface. These reefs are the danger of unwary mariners. The passages through them are intricate and narrow, and require all the skill of the native pilots to be navigated safely. The ship, threading some of these devious and dangerous channels, seems at times to be moving through beds of vegetation, so profuse is the growth under the water. All kinds of ferns and grasses, of weeds and of verdure, cushion and conceal the dangers of the reefs. The clearness of the water lessens in appearance its actual depth, and the bottom of the vessel and the bottom of the sea seem to be brought so nearly together that the jar of contact is at any moment apprehended. The danger, however, is more apparent than real, and few accidents happen to the small and easily handled craft that trade in those waters.

The islands are, in parts and places, of coralline or zoophyte formation, and are believed to be the

LIP OR RIM OF A CRATER, or the top of a mountain, thrown or lifted into position, or in some other way affected by volcanic action or matter in years long past. There are marks and signs which indicate the forces and effects of heat and fire; and shells and parts of shells, and other memorials of the sea, can be found imbedded in the soft stone, or mixed in with the sand, in almost any part of the islands. However formed, or under whatever pains and throes they came into existence, there the islands are, all by themselves, in the middle of the Atlantic Ocean, tiny strips of verdure in the clearest of water, as gay and as bright as if they were continents, and possessed hills and vales, rivers and lakes, and all the accessories of a first class, extensive and growing country. The orange and the lemon flourish in the land; the cedar and the palmetto crown each ridge, or line and hedge every road; from every height glimpses of the ocean, either in its seaward expanse or in its numerous bays and inlets, can be seen; and the tourist is everywhere gladdened by the summer warmth and placidity of the scene. Of course one cannot live by sight alone, and the lemon and orange even do not fill up the measure of all human taste or needs. But when the vital forces have been squandered or impaired in the whirl and hurry of busier places, or when the poor battered lungs can only wheeze and gasp with the thermometer at zero, it is delight and strength to bask and loiter in the soft, serene warmth and peace of these more tropical neighbors of ours.

THE SOIL OF BERMUDA

is most fertile. It produces year after year without the high manuring or long rests of other soils. The seaweed or kelp that everywhere floats in its bays and coves is the common fertilizer. It is the flotsam and jetsam of the waves, which all the planters have free access to, without tithe or toll. Two kinds of potato, the sweet and the white, and two crops of each, can be grown each year. The delicate white potato that comes to us in early spring from the Bermudas lacks the faculty which Mr. Toots valued so highly in his wife—it cannot repeat or reproduce itself. It is the poorest of propagators, and every year the common Mercers, or other potatoes of Ohio or Illinois, are sent to Bermuda to be planted, and in a few months come back to us the pink and thin skinned delicacies so much affected by the New York palate. The growing of vegetables for the markets of the United States is fast becoming the chief business of Bermuda. A few of the onions from there find their way to the West Indies, and the bulk of the arrowroot goes to London. But, with these exceptions, the garden produce comes this way, and in the spring, before our home markets send their supplies, the streets and the wharves of the Bermuda towns are awakened from their usual dulness and are crowded and bustling, and the supreme energy and effort of the year are displayed in packing, forwarding, and shipping their potatoes, onions, tomatoes and other delicacies to New York and other ports on the Atlantic seaboard.

There are two towns in Bermuda, Saint George and Hamilton —the latter being the larger and more important, the Governor residing and the Government officers being all situated there.

It is, because of its isolation and geographical bearings to this continent, an important dependency of the British Crown. It is largely owned, and is governed and garrisoned by the British Government. There are fortifications at either extremity and in the centre of the island; there is a dock yard, and there is also one of the largest floating marine slips or docks in the world. It was built in England, was sailed or floated to Bermuda by the help and under convoy of large war vessels, and now, in its safe resting place, can take and hold in its girth the largest iron-clads of the English navy. There are regiments of foot and companies of artillery and engineers, and all contrivances and appliances for fitting out ships and defending approaches. The Governor is appointed by the Crown, and so are the Executive and Legislative Council and most of the chief officers of the colony. But the House of Assembly is elected by the people, and there are thirty-six members to represent the nine parishes, comprising in all a population of some 12,000. The Colonial Parliament meets once a year, and is opened with much ceremony.

The visitor to Bermuda who seeks the pleasure and excitement of cities will be disappointed. If he does not enjoy boating and fishing; if he tires soon of the same walks and rides; if he does not need the compensations of warmth and convalescence; if his nature be altogether busy and restive, and not given to meditative moods, then, probably, his days will be tedious. There are no operas or theatres, none of the contrivances of larger cities for helping or improving time. The place is small, simple and unpretentious; the people are kind, hospitable, and unaffected in their tastes and habits. Good and sufficient food can always be had, and clean and comfortable lodgings, at prices that need not alarm people of moderate means—and this without any danger of fever and ague, and with mosquitoes fewer in numbers and less clamorous and violent in attack than their congeners of the State of New Jersey. There are no poisonous insects on the islands, no scorpions to make the drawing on of boots a danger, no centipedes to make getting into bed a terror. The worst, and only thing of the kind, is the cockroach, which, it must be said, is too fond of joining family circles at ea, and displays an enterprise and social disposition somewhat superfluous. But for the rest, one can get along well enough, and would find little cause to complain. Always near, and nearly always enjoyable, are the waters of the ocean, sparkling and glistening in numerous bays and harbors; the sky blue and bright, and wonderfully luminous at night with moon and stars; and the atmosphere, which brings to sound lungs pleasure, and to weak ones health and strength as well as warmth and purity. —*The Sanitarian.*

Graham Crackers.—J. A. Currier, proprietor of the well known cracker bakery, 436 Greenwich street, has sent us a box of Dr. Trall's celebrated Graham Crackers, and we have kept the package open for the benefit of callers. We think our friends have rather increased in number since we opened the box. The crackers appear to be made of fine white wheat, coarsely ground, or rather crushed, and possess the sweet flavor of the natural grain. They are, in fact, far more palatable than ordinary crackers, and more agreeable than any other compound made from unbolted wheat with which we are acquainted. The frequent use of these crackers must prove beneficial to a large class of dyspeptic persons, and should be encouraged in the young, who require the bone forming phosphates contained in the hull of grain, and which does not exist in superfine flour. The price of these choice articles of foot is from 12 to 14 cents per pound, according to quantity purchased.

